# Water homeostasis and extracellular fluid dynamics reveal imidacloprid disruption and rutin mitigation in honey bees

**DOI:** 10.1038/s41598-025-26833-0

**Published:** 2025-11-28

**Authors:** Juan P. Hernández, Fredy Mesa, Andre J. Riveros

**Affiliations:** 1 Departamento de Biología, Escuela de Ciencias E Ingeniería, Universidad del Rosario, Cra. 26 #63B-48, Bogotá, Colombia; 2 Facultad de Ingeniería y Ciencias Básicas, Fundación Universitaria Los Libertadores, Bogotá, Colombia

**Keywords:** Water homeostasis, Extracellular fluid evaporation, *Apis mellifera*, Imidacloprid, Rutin, Biological techniques, Biophysics, Ecology, Systems biology, Zoology

## Abstract

Pollinator decline is partly driven by exposure to agrochemicals such as imidacloprid, a neonicotinoid insecticide that impairs feeding and water balance in bees. This study examines how imidacloprid and the dietary flavonoid rutin, individually and combined, affect body mass regulation and extracellular fluid in *Apis mellifera*. Africanized honey bees were exposed to 0.3, 0.6, or 1.0 ppm of each substance. We conducted four experiments to evaluate short- and long-term changes in food intake, wet and dry mass, hydration, and fluid evaporation rates. Imidacloprid consistently reduced food consumption and body mass gain, while rutin promoted weight gain, increased water retention, and partially reversed imidacloprid’s effects. The combination treatment produced intermediate results. Notably, extracellular fluid from imidacloprid-treated bees showed significantly slower evaporation, suggesting altered physicochemical properties. This represents the first application of extracellular fluid evaporation as a physiological biomarker of sublethal stress in honey bees, expanding current approaches beyond conventional endpoints such as survival or behavior. These physiological changes persisted beyond the exposure period in some cases. Our results highlight the potential of mass dynamics and fluid behavior as sensitive markers of sublethal stress. Rutin appears to enhance physiological resilience, supporting its role as a candidate dietary mitigator. This work contributes to the development of new physiological endpoints for assessing the impact of agrochemicals on pollinators and offers insight into the protective role of plant-derived compounds.

## Introduction

Bees, particularly *Apis mellifera*, play a central role in pollination processes that sustain global agriculture and biodiversity^1,2^. Approximately 75% of crops consumed by humans benefit from animal pollination^3^, with bees being the most efficient and widespread pollinators. Their ecological and economic^4,5^ importance has prompted extensive research efforts aimed at identifying and mitigating threats to their health and survival^6,7^. However, despite their importance, bee populations have been declining due to a range of anthropogenic stressors^8^, including habitat loss^9^, climate change^2,10,11^, pathogens^12,13^, and especially agrochemical exposure^14–16^.

Among the principal agrochemical threats, neonicotinoid insecticides have gained particular attention^17–20^ due to their systemic nature and persistence in the environment. Imidacloprid, one of the most widely used neonicotinoids, acts by binding irreversibly to nicotinic acetylcholine receptors in insect neurons^21^, causing sustained depolarization and leading to motor dysfunction^22,23^, paralysis, or death^24^. Notably, sublethal concentrations can impair foraging behavior^25–28^, alter food consumption^29^, induce regurgitation, and compromise water homeostasis in bees^30,31^. Residues of imidacloprid have been detected in nectar and pollen of treated crops^32–34^, exposing foraging bees to chronic low doses with long-term physiological consequences^14,35^.

The regulation of water balance in insects is a tightly controlled physiological process involving ingestion, absorption^36^, evaporation, and osmoregulation^37–39^. Honey bees, like many terrestrial insects, rely on aquaporins and ionic pumps in the alimentary canal and excretory organs to regulate internal water content in response to environmental conditions^40^. Factors such as ambient temperature^41^, humidity^42^, and solute concentration in food sources affect these regulatory mechanisms. Any disruption to this system, whether through environmental change or chemical exposure, can impact hemolymph composition^43–45^, cellular function, and overall survival^46^. Given the high surface-area-to-volume ratio of bees and their continuous foraging activity, maintaining water balance is essential for thermoregulation, muscle performance, and neurophysiological stability^47,48^.

Recent studies have proposed that certain phytochemicals naturally found in nectar and pollen may offer protective effects against agrochemical stress^49–51^. Flavonoids, such as quercetin and its glycoside rutin^52^, exhibit antioxidant^53^, anti-inflammatory^52^, and neuromodulatory properties^54^. Rutin in particular has been associated with improved cognitive recovery^55^, reduced oxidative stress, and enhanced detoxification in bees exposed to pesticides^16,56,57^. In addition to modulating cellular responses to toxins, flavonoids may interact with membrane transporters^52,58^, antioxidant systems, and metabolic enzymes, potentially enhancing physiological resilience. Moreover, rutin is part of the natural diet of bees, suggesting it may be a biologically compatible compound for supplementation^59^.

Despite its promising properties, little is known about how rutin affects water balance, mass gain, or extracellular fluid dynamics in bees. Furthermore, no studies to date have assessed its potential to mitigate imidacloprid-induced physiological alterations, particularly those related to fluid regulation and mass variation. Addressing this gap is critical for understanding the full spectrum of sublethal pesticide effects and for identifying nutritional strategies that support pollinator health.

To our knowledge, extracellular fluid evaporation has received little attention as a biomarker for pesticide-induced physiological disruption in honey bees. Introducing this endpoint provides a novel approach to detect subtle alterations in water balance and homeostasis beyond conventional behavior and survival assays. The aim of this study is to evaluate the effects of imidacloprid and rutin administered individually and in combination on body mass, water content, and extracellular fluid properties in *Apis mellifera*. By focusing on physiological responses such as food intake, hydration, and fluid evaporation, this work seeks to establish quantitative markers for assessing sublethal chemical impacts on bee health and to explore the potential protective role of rutin.

## Materials and methods

### Experimental animals

Africanized honey bees (*Apis mellifera*) were collected from the apiary of Universidad del Rosario (Bogotá, Colombia). Foragers were randomly collected at the hive entrances from four source colonies. Since foragers represent a naturally mixed-age population of workers, our experimental groups included bees of variable age, which is standard in oral exposure assays. A total of 5035 bees were used across all experiments. Bees were housed in 250 mL disposable plastic containers, adapted for ventilation and substance administration. Prior to experiments, bees were maintained for 24 h under controlled conditions (temperature: 32 ± 6 °C; relative humidity: 51 ± 8%; light wavelength: 750 nm; which minimizes disruption during feeding assays, and fed ad libitum with a 1 M sucrose solution (31.1% w/w). After this habituation period, bees were randomly assigned to experimental groups. All procedures were conducted under constant temperature and humidity to minimize environmental variation. At the end of each trial, bees were sacrificed by exposure to − 30 °C for 24 h using a Thermo Scientific Revco Freezer (UGL3020A).

### Feeding containers and delivery system

Feeding systems were adapted from internationally recognized protocols for assessing oral toxicity in honey bees^60^. Each container was fitted with a feeding device consisting of a 40 mL glass bottle, a 1000 µL micropipette tip, and absorbent paper to regulate flow. The narrow end of the tip was inserted through the base of the container, ensuring continuous access to the test solution while minimizing spillage and evaporation. Containers were visually monitored during exposure to confirm active feeding behavior and solution depletion.

### Substances and dosages

Two substances were evaluated: the commercial insecticide imidacloprid (Confidor 350 CS, 350 g/L active ingredient) and the flavonoid rutin. Both were diluted in 1 M sucrose solutions to prepare three working concentrations: 0.3 ppm, 0.6 ppm, and 1.0 ppm. The solutions were labeled according to substance and concentration (e.g., 0.3Im, 0.6Ru). Solutions were prepared fresh on the day of each experiment. The concentration range (0.3, 0.6 and 1.0 ppm) was selected within the levels of imidacloprid residues detected in nectar and pollen under agricultural conditions. A comprehensive review reported median concentrations in nectar of 0.73mg/kg and mean values up to ~ 2.5 mg/kg^32^, with observed ranges from 6.0 × 10^–6^ to 20 mg/kg^33^. Modeling approaches further confirm that foraging bees are exposed to residuals typically within this range^34^. Experimental studies have also employed similar sublethal concentrations ranging from 0.1 to 10 ppm^30^. For rutin, concentrations were chosen according to its natural occurrence in nectar and pollen^56^ and its experimental use as a dietary supplement in pollinators^55^.

### Short-term exposure assay for food intake and body mass

Bees were exposed for 4.1 h to sucrose solutions containing 1.0 ppm of imidacloprid (Im), rutin (Ru), or both (Im + Ru). Twelve groups (approximately 71 bees per group) were monitored. Mass measurements of feeders and containers were taken every 15 min using a precision balance (Radwag AS 220.R2) to calculate food intake and body mass changes. Correlations between food intake and bee mass were analyzed, and treatment-specific differences were evaluated using linear regression and ANOVA.

### Dose- and time-dependent assay for hydration and body mass

Bees were exposed to ten treatments (0.3, 0.6, and 1.0 ppm of Im, Ru, and Im + Ru, plus control) across three times (1, 2, and 3 h). After exposure, bees were frozen, and groups were weighed for wet mass. Subsequently, they were dried at 70 °C for 3 days (Binder FED 720 oven) to determine dry mass and calculate water content. Each condition was replicated three times using separate containers and bee batches. Water content was estimated by subtracting dry mass from wet mass, and values were normalized per bee. Data were analyzed with factorial ANOVA, and post hoc Tukey HSD tests were used to evaluate group differences.

### Two-phase exposure assay for recovery of feeding and hydration

Bees were assigned to three groups (Im, Ru, and control) for 24 h of initial exposure (Phase 1), then subdivided and re-assigned to nine combinations for an additional 24 h (Phase 2). Each treatment combination was represented by two or three replicates, totaling 18 groups. Mass of ingested food and total bee wet and dry masses were recorded to evaluate cumulative and recovery effects on water content and satiety. This design enabled assessment of persistence and reversibility of physiological effects under chronic and sequential exposure scenarios.

### Extracellular fluid extraction and evaporation assay

**Fig. 1:**
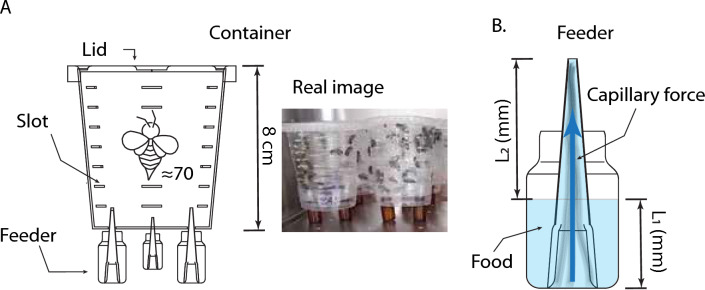
Experimental setup for bee confinement and feeding. **A)** Diagram of the plastic container used to house approximately 70 bees per group. The container includes lateral ventilation slots and three feeder ports at the base. A real image of the experimental groups is shown on the right. **B)** Schematic of the feeding system consisting of a 40 mL glass bottle and a micropipette tip. Capillary forces maintain a stable fluid column. L_1_ and L_2_ denote fluid column heights inside the bottle and tip, respectively.

After 24 h of exposure to 0.3 ppm Im, Ru, or Im + Ru, extracellular fluid was extracted from centrifuged bees^61^ following previously described protocols. For each replicate, 15 bees were cold-anesthetized, dissected, and placed in 1.5 ml microcentrifuge tubes. Centrifugation was performed at 3000 RCF for 5 min. Four independent replicates were obtained per treatment, resulting in 12 groups in total. Fluid samples were placed at 34.5 ± 0.4 °C, and their mass was measured every 10 min over 1 h using a Radwag AS 220.R2 balance. Each replicate was analyzed independently. Evaporation curves were fitted to exponential decay models to estimate evaporation coefficients (Fig. 1).

### Statistical analysis

All statistical analyses were conducted using RStudio (v4.3.1). Normality and homoscedasticity of residuals were verified via Shapiro–Wilk and Levene’s tests, respectively. When assumptions were not met, data transformations or non-parametric tests were applied. Statistical tests were selected according to the experimental design:

- Experiment 1 (short-term food intake and body mass): analyzed by regression and one-way ANOVA.
- Experiment 2 (wet and dry mass across treatments and time): analyzed by factorial ANOVA.
- Experiment 3 (two-phase recovery assays): analyzed by two-way ANOVA.
- Experiment 4 (extracellular fluid evaporation): analyzed by nonlinear regression (nls) fitted to exponential decay models; fitted coefficients were compared by ANOVA.

Tukey´s HSD was used for pairwise comparisons, and significance was established at α = 0.05.

## Results

### Experiment 1: short term effects on body mass and food intake

**Fig. 2:**
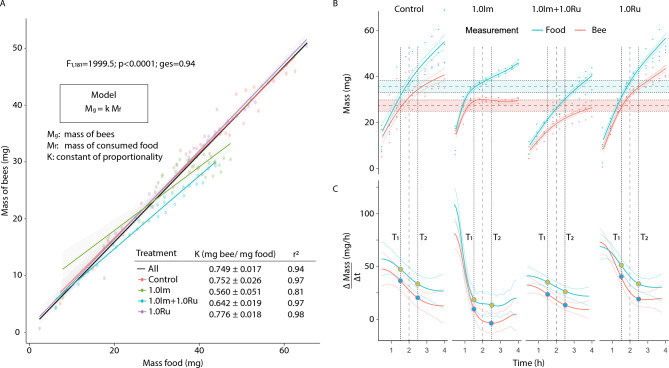
Relationship between food intake and body mass during short-term exposure (Experiment 1). **A)** Linear regression between total food consumed and average bee body mass after 4.1 h of exposure to four treatments. Shaded areas indicate 95% confidence intervals. **B)** Temporal evolution of food consumption and body mass for each treatment. Dots represent group means; lines indicate model fits and trends over time.

A total of 847 bees were randomly assigned to 12 groups (mean size: 70.6 ± 4.8 bees) exposed to 1.0Im, 1.0Ru, 1.0Im + 1.0Ru, or control. Bee body mass exhibited a strong positive correlation with food consumption (slope: 0.749 ± 0.017 mg bee/mg food; R^2^ = 0.94). However, the relationship differed significantly among treatments (F_1;118_ = 1999.5; p < 0.0001). Mass gain per milligram of consumed food varied across treatments (mean ± sd): 0.560 ± 0.051 mg bee/mg food (1.0Im), 0.642 ± 0.019 mg bee/mg food (1.0Im + 1.0Ru), 0.776 ± 0.018 mg bee/mg food (1.0Ru), and 0.752 ± 0.026 mg bee/mg food (Control) (Fig. 2A).

Consumption and body mass increased over time, but rates differed by treatment. At 2 h, food intake showed differences across treatments (mean ± sd): 25.2 ± 1.1mg (Im + Ru), 31.5 ± 1.1mg (Im), 34.6 ± 1.6mg (Ru), and 35.7 ± 2.6 mg (Control). The body mass also increased initially but plateaued for 1.0Im after 2 h, despite continued feeding: 17.1 ± 1.1mg (Im + Ru), 22.4 ± 1.1mg (Im), 26.0 ± 1.2 mg (Ru), and 27.3 ± 2.4 mg (Control) (Fig. 2B).

Rates of change in food consumption and bee body mass were analyzed during two intervals: T1 (1.4–1.6 h) and T2 (2.4–2.6 h). In the interval T1, both food consumption and body mass gain were lowest under imidacloprid exposure and highest under rutin (mean ± SD): Food_Im_ = 22.4 ± 2.2 mg/h; Bee_Im_ = 14.69 ± 3.00 mg/h; Food_Ru_ = 55.8 ± 1.3 mg/h; Bee_Ru_ = 47.4 ± 2.1 mg/h. In the interval T2, food consumption and body mass declined in the imidacloprid group, while the rutin group maintained moderate rates (mean ± SD): Food_Im_ = 13.9 ± 0.7 mg/h; Bee_Im_ = –3.5 ± 0.5 mg/h; Food_Ru_ = 35.9 ± 0.8 mg/h; Bee_Ru_ = 21.0 ± 0.5 mg/h. In the control group, rates remained relatively stable: Food_Control_ = 48.4 ± 0.8 mg/h and Bee_Control_ = 37.3 ± 1.0 mg/h in T1; Food_Control_ = 34.0 ± 0.7 mg/h and Bee_Control_ = 20.7 ± 0.8 mg/h in T2. These findings indicate that imidacloprid impairs body mass gain despite continued intake, whereas rutin supports sustained weight increase (Fig. 2C).

### Experiment 2: dose- and time-dependent effects on wet and dry mass

**Fig. 3:**
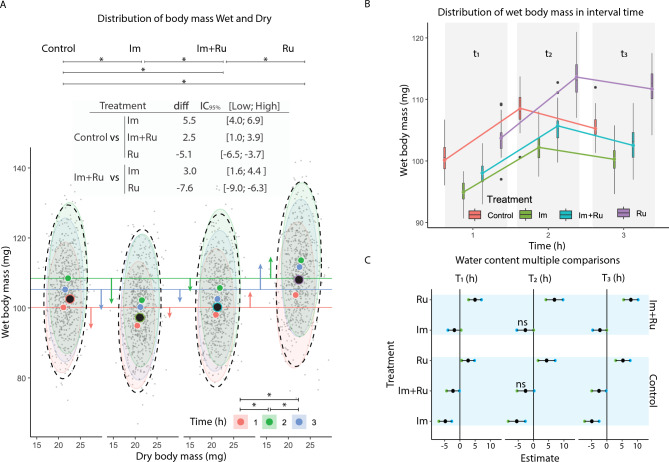
Effect of imidacloprid and rutin on wet and dry body mass (Experiment 2). **A)** Bivariate plot of dry vs. wet mass after 1, 2, and 3 h of exposure. Ellipses represent 95% confidence regions. Differences between treatments are indicated. **B)** Temporal profiles of wet mass (mg) for each treatment group. **C)** Water content estimates derived from paired dry/wet mass measurements. Groups labeled with different letters are statistically different (Tukey HSD, p < 0.05).

A total of 2652 bees were assigned to 36 groups (mean size: 73.7 ± 1.7 bees) exposed to ten treatments at three time points. Significant effects were observed for both wet and dry mass (F_Wet 3;2648_ = 129, p < 0.0001; F_Dry 3,2648_ = 51, p < 0.0001). Wet mass means (diff, 95% CI) were: 5.5 [4.0; 6.9] (Im), 2.5 [1.0; 3.9] (Im + Ru), –5.1 [–6.5; –3.7] (Ru). Im + Ru differed from Im (3.0 [1.6; 4.4]) and Ru (–7.6 [–9.0; –6.1]). Concentration had no effect (p > 0.05) (Fig. 3 A). Time had a significant effect on the wet mass (all p < 0.0001). Mass followed a non-linear pattern: it increased from t1 to t2 but decreased at t3 (Fig. 3 B). For Im: [94.9 ± 8.4; 102.2 ± 9.0; 100.3 ± 9.7], Im + Ru: [98.0 ± 8.8; 105.7 ± 10.5; 102.5 ± 9.4], Ru: [103.7 ± 10.0; 113.6 ± 10.5; 111.7 ± 9.3]. Water content estimates varied by treatment and time (p < 0.0001), with Im decreasing and Ru increasing it (Fig. 3 C).

### Experiment 3: two-phase feeding and physiological recovery

**Fig. 4:**
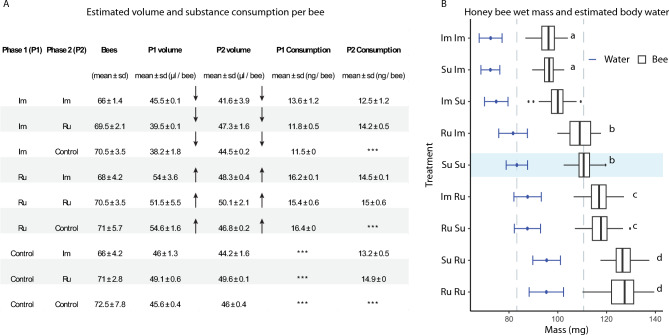
Consumption and body mass recovery after two-phase exposure (Experiment 3). **A**) Table summarizing consumption volume and estimated dose (mean ± sd) per bee across both phases. **B)** Boxplots of final wet mass and estimated water content per treatment. Blue segments show estimated water; white segments show body mass. Groups with different letters differ significantly.

A total of 1250 bees were organized into 18 groups (mean: 69.4 ± 3.9 bees). In Phase 1, bees consumed varying amounts of sucrose: lowest with Im (364–456 µL), highest with Ru (479–570 µL) (Fig. 4 A). During Phase 2, groups previously exposed to Im increased intake when switched to Ru. Im suppressed consumption, while Ru restored it. Wet mass differed significantly among treatments (F_8,1241_ = 1169.5, p < 0.0001). Lowest values were recorded for Im–Im and S–Im, with no difference between them (Diff = 0.2; 95% CI: [–1.4, 1.8]). Bees previously on Im but switched to sucrose recovered mass (99.6 ± 3.7 mg). Water content was lowest with Im (72.4 ± 4.3 mg) and highest with Ru (127.1 ± 4.7 mg). Switching from Im to Ru restored hydration (87.6 ± 5.3 vs. 81.6 ± 5.8 mg) (Fig. 4 B).

### Experiment 4: evaporation of extracellular fluid

**Fig. 5:**
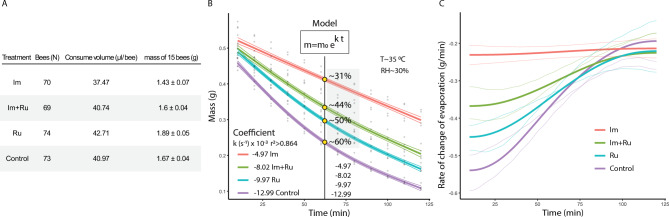
Evaporation dynamics of extracellular fluid (Experiment 4). **A)** Table with the number of bees, volume of sucrose solution consumed, and the initial combined mass of 15 bees per replicate across treatments. **B)** Evaporation curves fitted to exponential decay models. Percentages indicate relative reduction in evaporation compared to controls. **C)** Temporal dynamics of extracellular fluid evaporation over 60 min, with fitted exponential curves illustrating differences in decay rates among treatments.

A total of 286 bees were divided into 12 groups (mean: 71.5 ± 2.4 bees). During the 24 h exposure period, bees consumed different amounts of sucrose solution depending on treatment. Control bees consumed on average 40.97 µl per bee, while consumption was slightly lower in Im + Ru (40.74 µl) and higher in Ru (42.71 µl). imidacloprid-treated bees consumed the last solution (37.47 µl). The combined mass of 15 bees per replicate showed different masses across treatments (F_3,16_ = 13.5, p = 0.00367), with lower values in Im (1.43 ± 0.07 g) and higher values in Ru (1.89 ± 0.04 g), while Control (1.67 ± 0.04 g) and Im + Ru (1.60 ± 0.04 g) showed intermediate means (Fig. 5 A). These differences indicate that exposure affected both feeding and body condition prior to fluid extraction.

Mass loss during evaporation also differed significantly among treatments (F_3,188_ = 21.2, p < 0.0001), following an exponential decay.

Evaporation coefficients (k × 10⁻^3^ s⁻^1^) were –12.99 (Control), –9.97 (Ru), –8.02 (Im + Ru), –4.97 (Im). Compared to controls, extracellular fluid from Im-treated bees evaporated approximately 60% more slowly, while Ru-treated bees showed a ~ 23% reduction. The Im + Ru combination produced intermediate results (~ 38% reduction) (Fig. 5 B). These quantitative differences were consistent with the temporal dynamics of evaporation. Control and Ru samples exhibited the steepest initial decline within the first 30 min, reflecting higher evaporation rates. In contrast, fluids from Im- and Im + Ru-treated bees showed slower mass loss over time, indicating reduced evaporative performance. From 30 to 60 min, these differences became more pronounced: Ru remained close to Control, while Im consistently showed the flattest curve. The Im + Ru treatment was intermediate, evaporating faster than Im alone but slower than Control or Ru. Together, these results show that imidacloprid strongly reduces the evaporative potential of the extracellular fluid, whereas rutin maintains control-like dynamics and partially mitigates the disruption when co-administered with Im (Fig. 5 C).

## Discussion

This study demonstrates that the ingestion of imidacloprid and rutin, alone or in combination, produces measurable and divergent effects on body mass, water balance, and extracellular fluid properties in Africanized honey bees (*Apis mellifera*). The observed physiological alterations offer new insights into the impact of agrochemicals and dietary supplements on pollinator health, particularly in terms of water homeostasis and metabolic regulation.

### Imidacloprid reduces food intake and body mass gain

Across all experiments, exposure to imidacloprid (Im) consistently resulted in lower food intake and reduced body mass. In the short-term experiment (Exp. 1), bees exposed to Im showed a lower mass-to-food ratio and a plateau in weight gain despite continued consumption. This suggests impaired nutrient absorption or increased water loss (as shown in Fig. 2B). These findings are consistent with previous reports showing that imidacloprid impairs feeding behavior and induces regurgitation, which may explain the limited weight gain observed (Wong et al., 2018; Vergara-Amado et al., 2020). Importantly, the strong correlation between food intake and mass gain under control and Ru treatments (R2 > 0.95), contrasted with the disrupted relationship under Im, supports the hypothesis that imidacloprid affects not only ingestion but also internal processing of nutrients.

In the long-term exposure assay (Exp. 3), bees under continuous Im treatment maintained low hydration levels (wet mass: 72.4 ± 4.3 mg), and even after switching to sucrose, only partial recovery was observed (Fig. 4B). These data indicate that imidacloprid exerts persistent physiological stress, potentially via disruption of osmotic gradients or inhibition of aquaporin-mediated transport^40^. Furthermore, the reduced evaporation rate observed in extracellular fluid from Im-treated bees (k = –4.97 × 10⁻^3^ s⁻^1^; Fig. 5B) suggests compositional changes in the hemolymph, possibly due to altered ionic strength or protein content, which may impair normal water exchange and evaporation dynamics.

### Rutin enhances consumption, hydration, and recovery

In contrast, rutin (Ru) increased food intake and body mass in all experiments. During short-term exposure (Exp. 1), Ru-treated bees showed the highest consumption rates and consistent mass gain, supporting the idea that rutin promotes feeding behavior and efficient nutrient assimilation. In Exp. 2, Ru elevated both wet and dry body mass across all time points and concentrations, with statistical significance (p < 0.0001), indicating a robust physiological response. Moreover, in Exp. 3, bees previously exposed to Im and then transitioned to Ru partially recovered their hydration and food intake levels, with final wet mass values exceeding those of groups maintained on Im or switched to sucrose alone (Fig. 4B). This mitigative effect suggests that rutin enhances homeostatic recovery processes, potentially by reducing oxidative damage and restoring membrane integrity.

### Interactive effects and intermediate phenotypes with Im + Ru

The combination of Im and Ru produced intermediate values across multiple endpoints, including mass-to-food ratios, hydration, and evaporation dynamics. For example, in Exp. 4, the evaporation coefficient of the Im + Ru group (k = –8.02 × 10⁻^3^ s⁻^1^) was significantly different from both Im and Ru, suggesting a partial buffering of Im-induced changes in extracellular fluid composition. These results highlight the importance of evaluating not only isolated compounds but also their combined effects, as interactions can result in non-additive physiological outcomes.

### Evaporation as a sensitive marker of physiological disruption

The use of extracellular fluid evaporation as a physiological readout provides a novel perspective on internal water dynamics. Our findings show that hemolymph extracted from imidacloprid-treated bees evaporated more slowly than that from controls, while fluid from Ru-treated bees evaporated faster, resembling control levels. The inverse correlation between evaporation rate and imidacloprid exposure suggests that this parameter reflects shifts in fluid viscosity, osmolarity, or protein content. Each of these factors is relevant for thermoregulation and metabolic function. This marker could be valuable in future sublethal toxicity studies.

Slower evaporation in fluids from imidacloprid-treated bees may indicate increased viscosity or altered composition of the hemolymph^62^. This could reduce the efficiency of evaporative cooling. Impaired thermoregulation under these conditions may amplify pesticide toxicity^63,64^, as bees exposed to non-optimal temperatures exhibit higher mortality when challenged with imidacloprid^63^. Moreover, osmoregulatory capacity in honey bees depends on hemolymph osmotic balance, which is disrupted under desiccation stress^65^. Conversely, faster evaporation observed in rutin-treated bees suggests restoration of normal physicochemical properties of the fluid, supporting its role in maintaining homeostasis balance. Sublethal imidacloprid has also been shown to disrupt metabolic pathways in the hemolymph, reducing energy-related metabolites such as ATP and acetyl-CoA^62,66^, which may further influence the fluid´s physicochemical behavior.

### Limitations and future directions

While this study provides valuable physiological insights under controlled laboratory conditions, it has some limitations. The artificial environment cannot fully replicate field conditions where bees face multiple stressors simultaneously. Moreover, molecular mechanisms underlying the observed physiological changes were not characterized, and only worker bees were tested. Future studies should extend to other castes and pollinator species, as well as integrate molecular and proteomic approaches. Importantly, the evaporation rate of extracellular fluid may serve as a practical physiological marker in field monitoring and regulatory assessments, offering a novel tool for ecotoxicological evaluation.

### Implications and relevance

These findings collectively highlight that mass variation and fluid dynamics are sensitive physiological endpoints for detecting sublethal effects of agrochemicals. Unlike conventional behavioral or mortality assays, the measurement of water balance and evaporation offers a more nuanced perspective on internal physiological stress. The modulation of these parameters by dietary rutin reinforces its potential as a mitigative agent.

Given the growing concern over pollinator decline, especially in tropical environments where thermal and osmotic stresses are high, this study contributes to the broader understanding of how chemical exposures and dietary supplements interact to shape pollinator resilience. Future studies should explore the molecular basis of these effects, such as gene expression changes in water transport proteins, antioxidant enzymes, and membrane components. Additionally, field validation under natural foraging conditions would strengthen the ecological applicability of these results.

Overall, this work supports the integration of physiological biomarkers, such as mass variation and extracellular fluid behavior, into risk assessment frameworks for pollinators exposed to agrochemical mixtures.

## Conclusion

This study provides compelling evidence that exposure to imidacloprid and rutin modulates physiological traits directly related to body mass, water content, and extracellular fluid behavior in *Apis mellifera*. We demonstrated that imidacloprid impairs food intake, disrupts the relationship between consumption and mass gain, suppresses hydration, and alters the physicochemical properties of extracellular fluid. These effects were evident in both short-term and long-term exposures, and persisted even after cessation of treatment in some cases.

In contrast, rutin consistently promoted higher food intake, enhanced mass gain, increased water retention, and facilitated partial recovery from imidacloprid-induced stress. The intermediate results observed in the combination group (Im + Ru) highlight the complex interactions that can occur when both substances are present, suggesting that rutin may exert protective effects without fully counteracting imidacloprid toxicity.

Importantly, the inclusion of evaporation rate as a physiological readout provided a novel and quantitative measure of internal fluid dynamics, revealing sublethal disruptions not captured by conventional endpoints. These findings support the integration of fluid-based physiological markers into ecotoxicological evaluations of pollinators.

Our results emphasize the need for more comprehensive assessments of pesticide impacts.

These assessments should consider both ingestion and internal physiological consequences. They also point to the potential role of natural dietary supplements such as rutin in mitigating agrochemical stress. Future research should expand on these findings by incorporating molecular analyses and validating physiological markers in field-relevant conditions. Ultimately, this work advances the use of physiological indicators such as mass variation, hydration status, and extracellular fluid behavior as sensitive tools for monitoring pollinator health and resilience in agricultural environments.

## Data Availability

Data is provided within the manuscript.
